# Impact of Developmental Age, Necrotizing Enterocolitis Associated Stress, and Oral Therapeutic Intervention on Mucus Barrier Properties

**DOI:** 10.1038/s41598-020-63593-5

**Published:** 2020-04-21

**Authors:** Jaclyn Y. Lock, Taylor L. Carlson, Yueyue Yu, Jing Lu, Erika C. Claud, Rebecca L. Carrier

**Affiliations:** 1 Department of Bioengineering, Northeastern University, Boston, Massachusetts, USA; 2 Department of Chemical Engineering, Northeastern University, Boston, Massachusetts, USA; 3 Department of Pediatrics, Section of Neonatology, University of Chicago, Chicago, Illinois USA; 4 Department of Medicine, Section of Gastroenterology, University of Chicago, Chicago, Illinois USA

**Keywords:** Permeation and transport, Bacteria, Gastrointestinal diseases, Gastrointestinal models

## Abstract

Necrotizing enterocolitis (NEC) is a devastating gastrointestinal disease of incompletely understood pathophysiology predominantly affecting premature infants. While NEC is associated with microbial invasion of intestinal tissues, and mucus modulates interactions between microbes and underlying tissues, variations in mucus barrier properties with NEC-associated risk factors have not been investigated. This study explored differences in mucus composition (total protein, DNA, mucin content, sialic acid, and immunoregulatory proteins), as well as structural and transport properties, assessed by tracking of particles and bacteria (*E. coli* and *E. cloacae*) with developmental age and exposure to NEC stressors in Sprague Dawley rats. Early developmental age (5 day old) was characterized by a more permeable mucus layer relative to 21 day old pups, suggesting immaturity may contribute to exposure of the epithelium to microbes. Exposure to NEC stressors was associated with reduced mucus permeability, which may aid in survival. Feeding with breastmilk as opposed to formula reduces incidence of NEC. Thus, NEC-stressed (N-S) rat pups were orally dosed with breastmilk components lysozyme (N-S-LYS) or docosahexaenoic acid (N-S-DHA). N-S-LYS and N-S-DHA pups had a less permeable mucus barrier relative to N-S pups, which suggests the potential of these factors to strengthen the mucus barrier and thus protect against disease.

## Introduction

Necrotizing enterocolitis (NEC) occurs primarily in premature and low birth weight infants (<1500 g), and is associated with a 15–30% mortality rate^1,2^. Infants with NEC present with signs of mucosal inflammation in the distal ileum and proximal colon, bloody stool, abdominal distention, and respiratory distress. Depending on disease severity, treatment options include bowel rest, antibiotics, and/or emergency surgical procedures. Children who survive surgical intervention often face severe complications including neurodevelopmental delays^3,4^ and long-term neurological and intestinal rehabilitative needs^5,6^. The annual cost of caring for infants with NEC is estimated at $500 million to $1 billion.

Despite decades of research, the pathophysiology of NEC is still not completely understood^7–11^. NEC has been linked to inappropriate immune response, increased epithelial permeability, and altered microbiome^8,12–16^. Given the role of microbes in the pathogenesis of NEC, and the central role of intestinal mucus in controlling interactions between commensal bacteria and underlying tissues, we hypothesized that differences in the intestinal mucus barrier associated with immaturity may contribute to development of NEC.

Mucus is a dynamic barrier that is continuously secreted, degraded by the microbiome (e.g., mucin sugar cleavage)^17^, and shed from the mucosal surface^18^. The mucus barrier is composed of mucin glycoproteins, proteins, and lipids that covalently and non-covalently interact, creating a mesh-like structure with pores ranging in size from 10–500 nm^18–20^. MUC2, the main structural component of the intestinal mucus barrier, is heavily glycosylated with sugars that bind microbes, preventing their penetration to the epithelium^21,22^. Changes to the protective mucus layer, the body’s first line of defense at the mucosal epithelia, can alter exposure of the underlying epithelium to foreign antigens. It was recently demonstrated that mice with Hirschspung’s disease (HD), a developmental abnormality of the enteric nervous system associated with enterocolitis, had altered mucus barrier properties, as revealed by multiple particle tracking (MPT) in colonic mucus, compared to wild type control animals^23^. MPT is a nondestructive technique in which the trajectories of particles within a medium of interest, such as the cell cytoplasm or mucus, are analyzed to extract information about the local microenvironment^24–30^. Changes in mucus barrier properties in HD may play a role in the development of colonic inflammation. MPT was also utilized to show that physiological stimuli can affect transport properties and alter structure of mucus. Yildiz *et al*. reported that the presence of lipids (e.g., soybean oil) and calcium significantly decreased particle diffusion through and penetration of intestinal mucus *in vitro* and *in vivo*^31^. These results indicate that the mucus barrier can be altered in disease and in the presence of exogenous (e.g., orally dosed) stimuli.

Feeding with breastmilk as opposed to formula is a proven method to protect against development of NEC. Premature infants solely fed breast milk were 6–10 times less likely to develop NEC compared to infants exclusively fed formula^32^, suggesting breast milk components may play a beneficial role in protecting the immature gut. Breast milk contains multiple components (e.g., secretory lysozyme, polyunsaturated fatty acids, immunoglobulins, oligosaccharides, and platelet-activating factor^10^) that aid in mucosal integrity and immune function. However, in infant formula these components are absent or are present in low concentrations. We recently utilized MPT and time-lapse video microscopy of diffusing particles and swimming microbes to demonstrate that two breastmilk components, lysozyme (LYS) and docosahexaenoic acid (DHA), alter mucus barrier properties (i.e. decrease particle and microbe transport through healthy porcine intestinal mucus (data not published)). This finding motivates exploration of the impact of oral dosing of these agents on mucus barriers in rat pups exposed to NEC stressors.

Understanding changes in structure, composition, and transport properties of the mucus layer in disease states could motivate the development of therapeutic or prophylactic treatments to modulate the mucosal barrier. To test the hypothesis that developmental age impacts mucus barrier properties and could contribute to development of NEC, MPT and video microscopy were used to analyze both passive diffusion of particles and active swimming of microbes associated with NEC through intestinal mucus on tissue from early developmental age (5 day old) and weaned (21 day old) rat pups. In addition, mucus barrier properties on tissue harvested from pups exposed to NEC stressors (e.g., delivery one day prior to natural birth by cesarean section and exposed to hypoxia, formula feeding, and bacterial colonization) and surviving to 5 days of age were investigated in order to identify potential mucus barrier differences associated with survival during exposure to NEC stressors. Finally, LYS and DHA were orally dosed to pups exposed to NEC stressors to test their ability to “strengthen” mucus barriers in this disease model. Results supported dependence of mucus barrier transport properties, structure, and composition on developmental age and exposure to NEC stressors. Moreover, orally dosed LYS and DHA inhibited *Escherichia coli* transport significantly in intestinal mucus of pups exposed to NEC stressors.

## Results

### Transport properties of the protective mucus barrier are impacted by developmental age, exposure to NEC stressors, and oral dosing of LYS and DHA

The impact of developmental age (5do, 21do), exposure to NEC stressors (N-S), and prophylactic treatments (N-S-LYS, N-S-DHA) on intestinal mucus barrier properties was studied by analyzing the diffusion of particles (amine-, carboxyl-, and PEG- modified) and transport of bacteria (*E. coli* and *E. cloacae*) through an intact mucus layer on excised tissue. Analysis of passive particle diffusion provides an indication of changes in transport properties which may reflect altered structure or molecular interactions between diffusing entities and mucus components. Mobility of *E. coli* and *E. cloacae* in mucus is of interest as these bacteria have been associated with infection of mucosal tissue in NEC.

#### Mucus from an early developmental age (5do) provides a less significant barrier to passive diffusion relative to 21do intestinal mucus

**Figure 1:**
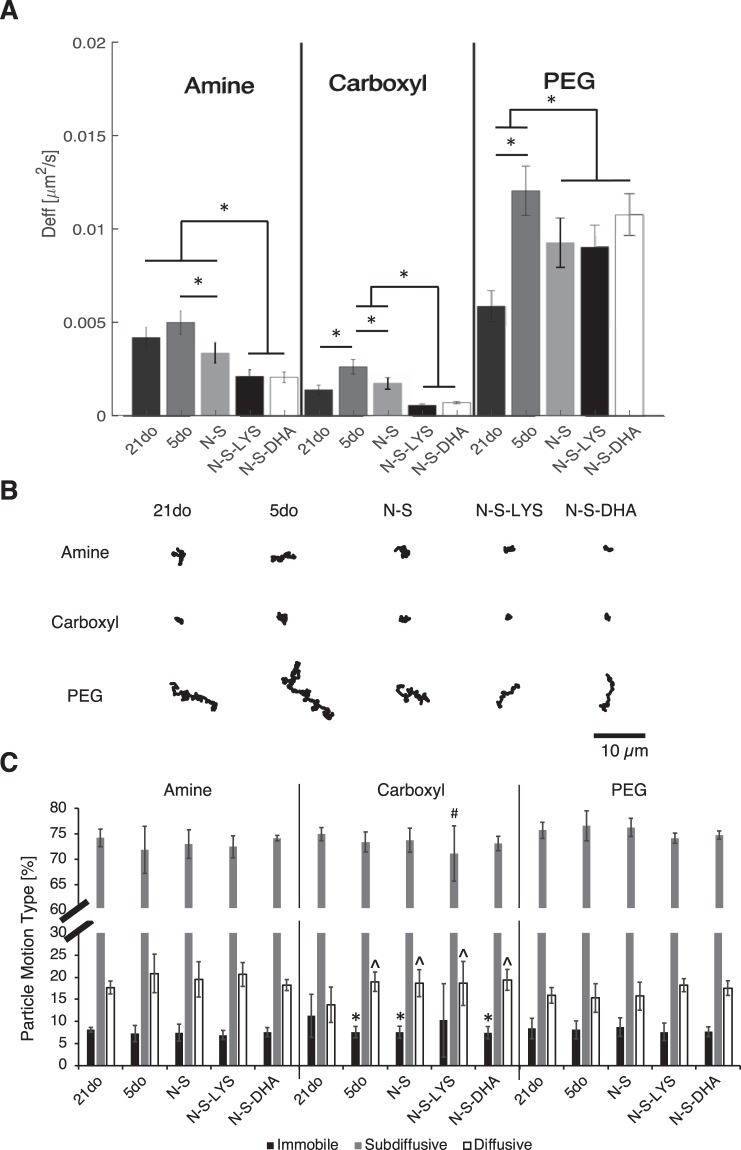
**(A)** Amine, Carboxyl, and PEG- modified particle effective diffusivity (Deff) on 21do, 5do, NEC-stressed (N-S), NEC-stressed and orally dosed with LYS (N-S-LYS), and NEC-stressed and orally dosed with DHA (N-S-DHA) tissue, at a time scale of τ = 1 sec. Data are presented as the average of at least 300 particles and error bars represent standard error of the mean of all tracked particles. ANOVA was used to determine significance between groups at *p < *0.05*. **(B)** Representative particle trajectories that were used to calculate Deff. Scale bar: 10 µm. **(C)** Particle motion type was characterized as immobile (α: 0–0.2), subdiffusive (α: 0.2–0.8), or diffusive (α: 0.8–1.0) by fitting the log-log MSD plot to find the anomalous exponent, α. Data are presented as the average of at least 300 particles and error bars represent standard deviation of all tracked particles. ANOVA was used to determine significance at *p < 0.05 compared to immobile carboxyl- modified particles on 21do, ^#^*p* < 0.05 compared to subdiffusive carboxyl- modified particles on 21do, ^*p* < 0.05 compared to diffusive carboxyl- modified particles on 21do.

Particles had enhanced mobility in mucus on tissue of an earlier developmental age (5do) (Fig. 1). Particles on 5do tissue had greater diffusivities (Fig. 1A) and visibly less confined trajectories (Fig. 1B) relative to 21do tissue. Particle diffusion through intestinal mucus was dependent on particle surface functionalization (amine-, carboxyl-, and PEG- modified) as well as tissue source (5do vs 21do) (Fig. 1A). PEG- modified particles, which have been previously characterized as non-interacting ‘stealth’ particles^27^, diffused the fastest and were found to penetrate and diffuse in between villi within 10 mins (Fig. S1). Amine- and carboxyl- modified particles diffused slower than PEG- modified particles and were observed to diffuse through mucus near villus tips after ~10 mins of adding particles, with positively-charged amine- modified particles diffusing faster than negatively-charged carboxyl- modified particles. There were similar percentages of immobile, subdiffusive, and diffusive amine- and PEG- modified particles tracked on all tissues (Fig. 1C). Carboxyl- modified particles had a significantly higher percentage of immobile particles and lower percentage of diffusive particles on 21do compared to 5do tissue.

#### Exposure to NEC stressors and oral dosing with LYS and DHA result in a less permeable mucus barrier

Survival after exposure to NEC stressors was associated with reduced mobility of particles regardless of surface functionality compared to same-age animals not exposed to stressors (5do). The mucus barriers of N-S-LYS and N-S-DHA pups were less permeable, as reflected by the significantly lower effective diffusivities of amine- and carboxyl- modified particles with visibly confined trajectories compared to N-S and 5do (Fig. 1A,B). The diffusion of PEG particles on N-S-LYS or N-S-DHA tissue was significantly hindered compared to 5do, but similar to N-S. There was a 13% increase in the survival of N-S-LYS pups compared to N-S alone and N-S-DHA (data not shown).

#### Developmental age, exposure to NEC stressors, and oral dosing of LYS and DHA all impact microbe speed in intestinal mucus

**Figure 2:**
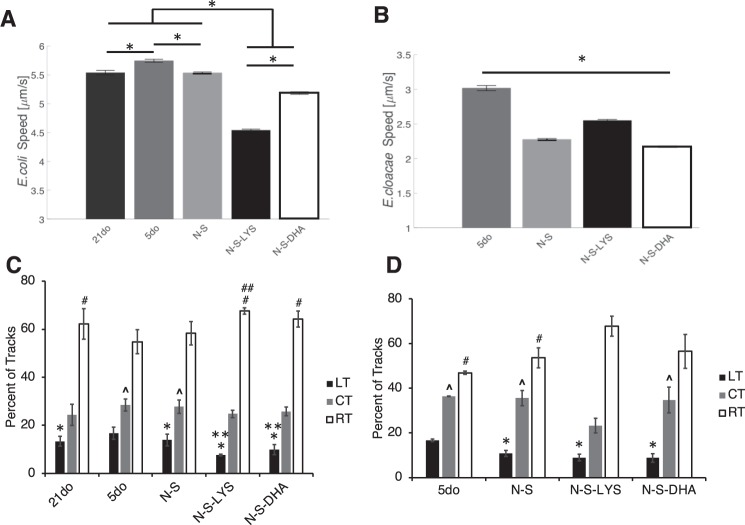
Average **(A)** *E. coli* and **(B)** *E. cloacae* speed on 21do, 5do, NEC-stressed (N-S), NEC-stressed and orally dosed with LYS (N-S-LYS), and NEC-stressed and orally dosed with DHA (N-S-DHA) tissue. Data are represented as the average of at least 300 microbes and error bars represent SEM. ANOVA was used to determine significance among groups with *p < 0.05. **(C)** *E. coli* and **(D)** *E. cloacae* microbe tracks were characterized as linear (LT), curvilinear (CT), or rotating (RT). ANOVA was used to determine significance in figure (C), LT: *p < 0.05 compared to 5do, **p < 0.05 compared to N-S; CT: ^*p* < 0.05 compared to 21do; RT: ^#^*p* < 0.05 compared to 5do, and ^##^*p* < 0.05 compared to N-S and 21do; and in figure (D), LT: *p < 0.05 compared to 5do; CT: ^*p* < 0.05 compared to N-S-LYS; RT: ^#^*p* < 0.05 compared to N-S-LYS.

Microbe tracking was used to analyze the movement of *E. coli* and *E. cloacae* in mucus on 21do (*E. coli* only), 5do, N-S, N-S-LYS and N-S-DHA intestinal segments. *E. coli* moved faster on 5do tissue (5.75 ± 0.03 μm/s, average ± standard error) than on 21do (5.54 ± 0.04 μm/s) (Fig. 2A). *E. coli* tracked on 5do tissue also had a higher percentage of linear tracks (17%) (Fig. 2C) and a lower percentage of rotating tracks relative to 21do.

Microbes on 5do tissue had faster speeds (*E. coli*: 5.75 ± 0.03 μm/s and *E. cloacae*: 3.01 ± 0.04 μm/s) than on N-S tissue (*E. coli*: 5.53 ± 0.02 μm/s and *E. cloacae*: 2.27 ± 0.01 μm/s) (Fig. 2A,B). *E. coli* speed in mucus on N-S-LYS (4.54 ± 0.02 μm/s) and N-S-DHA (5.19 ± 0.02 μm/s) was significantly decreased 1.22-fold and 1.07-fold, respectively, compared to N-S. There was also a significant decrease in *E. coli* tracks characterized as linear on N-S-LYS and N-S-DHA compared to N-S. *E. cloacae* speed in mucus on N-S-LYS was significantly increased (2.55 ± 0.01 μm/s), while it was significantly decreased on N-S-DHA (2.17 ± 0.01 μm/s), compared to N-S alone.

### Mucus composition varies with developmental age and exposure to NEC stressors

Developmental age and exposure to NEC stressors impact chemical composition of mucus collected from 21do, 5do, N-S, N-S-LYS and N-S-DHA pups.

#### Lower amounts of total protein, mucin, and DNA are found in early developmental age mucus

**Figure 3:**
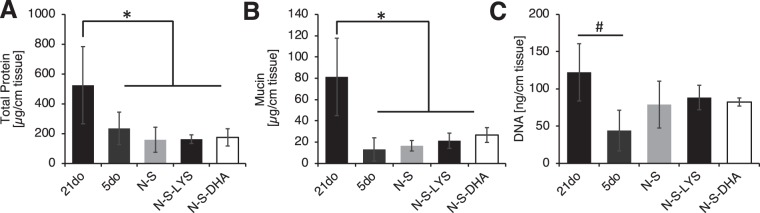
Mucus was collected from 21do, 5do, NEC-stressed (N-S), NEC stressed and orally dosed with LYS (N-S-LYS), and NEC-stressed and orally dosed with DHA (N-S-DHA) tissue and analyzed to quantify **(A)** total protein, **(B)** mucin, and **(C)** DNA amounts per length of tissue. Data are presented as average amount for at least 3 rats, and error bar represented as standard deviation. ANOVA was used to determine significance at *p < 0.05 comparing 21do to 5do, N-S, N-S-LYS, and N-S-DHA; and ^#^*p* < 0.05 comparing 21do and 5do.

Mucus collected from pups representing early developmental age (5do) had statistically lower amounts of mucin, total protein (normalized to length of intestine), and DNA than mucus from 21do pups (Figs. 3A–C and S2A DNA normalized to collected mucus amount). Mucus from N-S pups, with or without oral dosing of LYS or DHA, was similar with respect to total protein, mucin, and DNA amounts relative to 5do tissues.

#### Developmental age and exposure to NEC stressors impact amounts of immunoregulatory proteins

**Figure 4:**
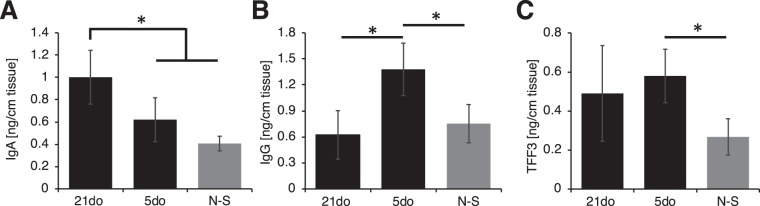
**(A)** Immunoglobulin A (IgA), **(B)** Immunoglobulin G (IgG), and **(C)** Trefoil factor 3 (TFF3) amount per length of tissue collected from 21do, 5do, and NEC-stressed (N-S) tissue. ANOVA was used to determine significance at *p < 0.05.

The amounts of TFF3 and immunoglobulins (IgA and IgG) were quantified per length of tissue (Fig. 4) and per volume of mucus collected (Fig. S2), as these proteins help maintain the integrity of the mucosa and protect the epithelium from foreign pathogens. Specifically, IgA aids in agglutination, entrapment of microbes in mucus, and clearance through peristalsis^33^, while IgG promotes the killing of microbes and clearance by macrophages and neutrophils^34^. TFF3 is known to be significant in mucosal protection and healing^35^. In collected mucus, the amount of IgA was significantly lower for 5do compared to 21do, both in terms of amount per length of intestine (Fig. 4A) as well as per volume of mucus collected (Fig. S2B). IgG amount, however, was significantly higher for 5do compared to 21do. TFF3 amount per length of intestine was similar for 5do and 21do (Fig. 4B,C). Mucus from N-S pups contained significantly lower amounts of IgG and TFF3 compared to 5do, while the amount of IgA was similar.

#### Exposure to NEC stressors results in lower sialic acid amounts in mucus

**Figure 5:**
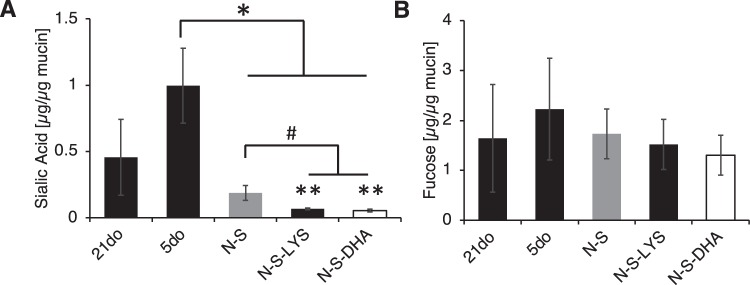
**(A)** Sialic acid and **(B)** fucose amounts in mucus collected from 21do, 5do, NEC-stressed (N-S), NEC-stressed and orally dosed with LYS (N-S-LYS), and NEC-stressed and orally dosed with DHA (N-S-DHA) tissue. ANOVA was used to determine significance at *p < 0.05 compared to 5do, ^#^*p* < 0.05 for N-S-LYS and N-S-DHA compared to N-S, and **p < 0.05 for N-S-LYS and N-S-DHA compared to 21do.

Mucin sugars fucose and sialic acid play an important role in bacterial binding. Mucin sugar amount was normalized to mucin amount to determine relative amount of sugar per mucin molecule. While there was not a statistically significant difference in sialic acid content with developmental age (5do vs 21do), exposure to NEC stressors resulted in significantly decreased sialic acid content relative to 5do mucus (Fig. 5A). Sialic acid content of N-S-LYS and N-S-DHA was further reduced relative to N-S alone. There was no statistical difference in fucose amounts among the groups (Fig. 5B).

### Structural features of intact mucus layer on 21do, 5do, N-S, N-S-LYS, and N-S-DHA tissue

Confocal and electron microscopy were utilized to analyze impact of developmental age, exposure to NEC stressors, and orally dosed treatments on mucus structure.

#### Confocal microscopy of non-fixed intestinal samples

**Figure 6:**
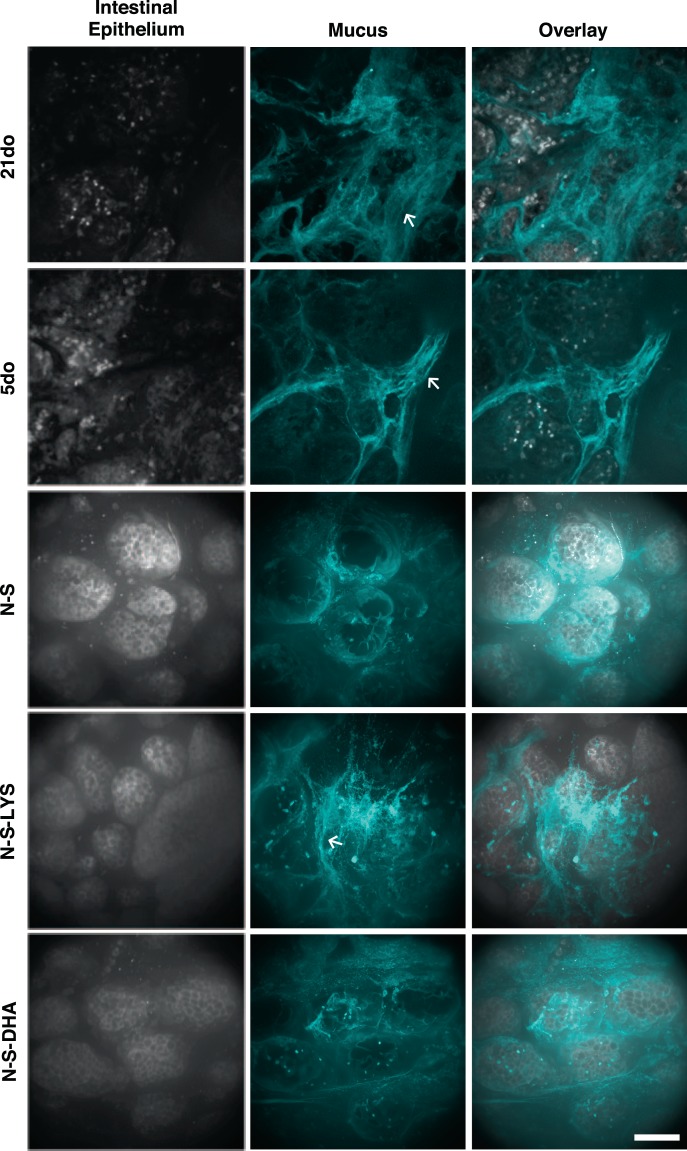
21do, 5do, NEC-stressed (N-S), NEC-stressed and orally dosed with LYS (N-S-LYS), and NEC-stressed and orally dosed with DHA (N-S-DHA) tissue was stained with DAPI showing cell nuclei (gray; 5do and 21do) or CellTrace^TM^ Bodipy^TM^ (gray; N-S, N-S-LYS, and N-S-DHA), and mucus (cyan) was stained with wheat germ agglutinin which binds sialic acid and *N*-acetylglucosaminyl residues. Striated (←) mucus structure is noted. Scale bar: 20 µm.

To visualize differences in mucus amounts and distribution, non-fixed tissue samples were stained with fluorescent wheat germ agglutinin (WGA), which binds to sialic acid and N-acetylglucosaminyl sugars (Fig. 6). Both 21do and 5do had striated mucus spread over the epithelium, however 21do had a visibly greater amount of mucus relative to other groups, with substantial staining of mucus across villi tips. N-S pups had altered mucus distribution and appearance compared to 5do. Mucus in N-S and N-S-DHA samples was clustered mainly around intestinal villi rather than across their tips, with N-S-DHA samples presenting a subtly more diffuse, homogenous staining pattern across villi. Mucus on N-S-LYS samples appeared to have a different staining pattern, with more intensely stained strands of mucus, potentially reflecting clustered mucin molecules, relative to other groups.

#### SEM micrographs of mucus coverage on tissue

Due to the highly aqueous nature of the mucus gel, conventional aqueous-based fixatives (e.g., formaldehyde) do not properly preserve and can collapse the native mucus structure^36,37^. Thus, two different non-aqueous fixatives^36,37^, Carnoy’s and perfluorohexane/1% osmium tetroxide, were utilized to fix the mucus layer.

**Figure 7:**
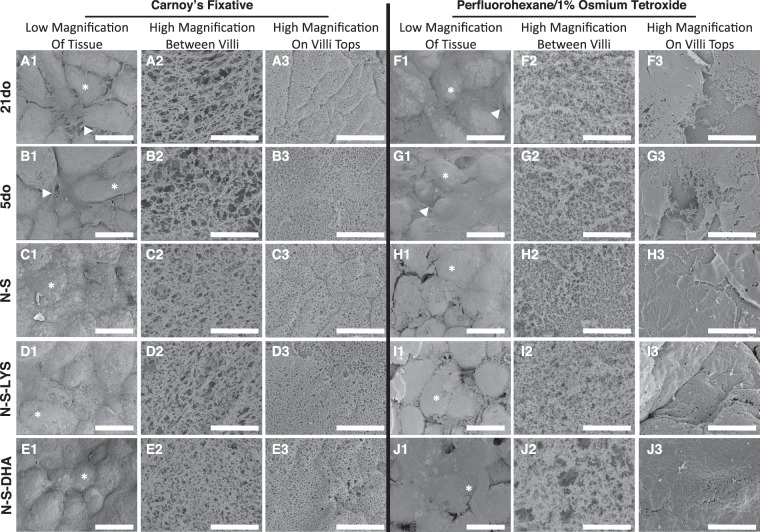
SEM micrographs of 21do, 5do, NEC-stressed (N-S), NEC-stressed and orally dosed with LYS (N-S-LYS), and NEC-stressed and orally dosed with DHA (N-S-DHA) intestinal samples fixed with Carnoy’s fixative (A-E) or perfluorohexane/1% oxmium tetroxide (F-J). Low magnification micrographs showing mucus distribution (A1-J1, scale bar = 100 µm) and high magnification micrographs showing mucus between villi (A2-J2, scale bar = 5 µm) and mucus on villi tops (A3-J3, scale bar = 5 µm). Villus head (*), and filamentous mucus between villi (▶) are noted.

Mucus coverage and microstructure appeared similar regardless of developmental age (21do vs 5do) (Fig. 7). In low magnification micrographs of 21do (Fig. 7A1,F1) and 5do (Fig. 7B1,G1) tissues, mucus did not form a continuous sheet, and mucus between villi was filamentous. While a majority of the 5do tissue had a non-continuous mucus sheet, there were a few instances of continuous mucus coverage. Although mucus microstructure was similar between 21do and 5do when visualized at high magnification, there were slight differences depending on the fixative used. When fixed with Carnoy’s fixative, mucus between villi had a globule-bearing filamentous structure with pores of varying size and visibly distinct mucin fibers (Fig. 7A2,B2). In comparison, perfluorohexane/1% osmium tetroxide fixative resulted in an agglomerated porous mucus structure (Fig. 7F2,G2). On villus tops, there was continuous mucus coverage with small pores (Fig. 7A3,B3) or a discontinuous mucus covering with compact structure (Fig. 7F3,G3) when fixed with Carnoy’s or perfluorohexane/1% osmium tetroxide, respectively.

Pups exposed to NEC stressors had visibly apparent differences in mucus coverage and microstructure. N-S tissues appeared to have a more continuous layer of mucus covering villi tops than 5do (Fig. 7C1,H1). When fixed with Carnoy’s fixative, mucus between villi was more aggregated and had smaller pores compared to 5do mucus (Fig. 7C2).

Tissues from N-S-LYS and N-S-DHA pups appeared to have a continuous mucus coverage similar to N-S (Fig. 7D1,E1). In low magnification micrographs, there were clusters of agglomerated mucus between villi in N-S-LYS tissue while the mucus covering appeared more continuous in N-S-DHA tissue, when fixed with perfluorohexane/1% osmium tetroxide (Fig. 7I1,J1). Moreover at high magnification, mucus between villi and on villus tops were similar between N-S-LYS and N-S alone, while N-S-DHA tissue had larger pores and visible single mucin fibers when compared to N-S alone fixed with perfluorohexane/1% osmium tetroxide (Fig. 7J2).

### TEM structural analysis

**Figure 8:**
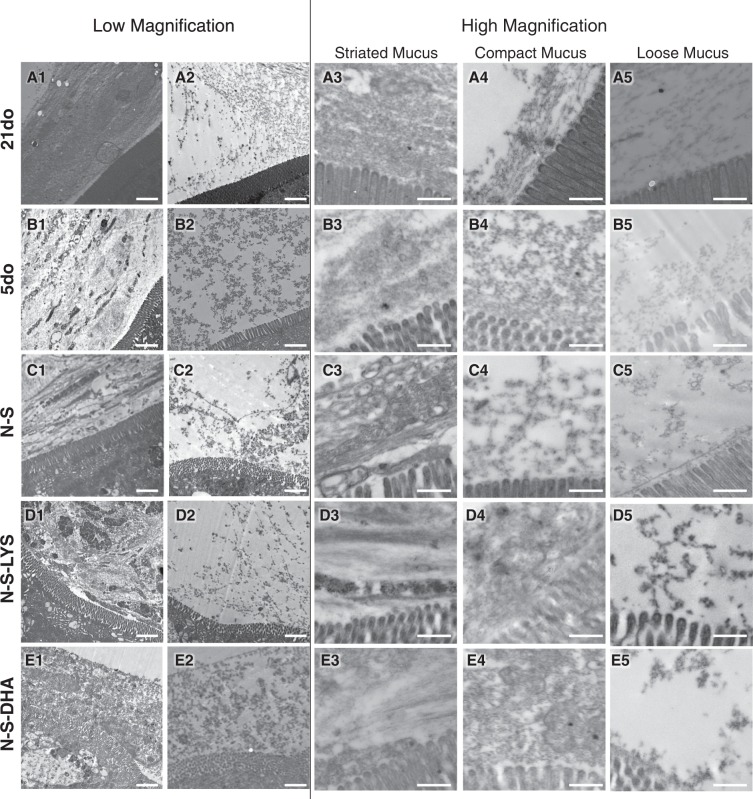
TEM micrographs of 21do, 5do, NEC-stressed (N-S), NEC-stressed and orally dosed with LYS (N-S-LYS), and NEC-stressed and orally dosed with DHA (N-S-DHA) ileal sections. Low magnification micrographs (A1-E2, scale bar = 2 µm), and high magnification micrographs of striated, compact, and loose mucus (A3-E5, scale bar = 500 nm).

Tissue samples were fixed as an intact intestinal tube and any food bolus present within the segment was not removed. Low magnification TEM micrographs revealed a heterogeneous mucus structure in all groups (Fig. 8). Specifically, in instances where food boluses were observed in the lumen, compact, striated mucus was observed adjacent to these boluses (Fig. 8A1–C1), while loose, unorganized mucus was found in between villi and adjacent to food boluses (Fig. 8A2–C2). Fewer instances of food boluses were noted in pups exposed to NEC stressors (N-S, N-S-LYS, and N-S-DHA) compared to 5do. All samples had areas of loose, compact, and striated mucus at high magnification (Fig. 8A3–E5).

## Discussion

NEC is a devastating disease that affects premature and low birth weight infants. Potential abnormalities in the mucus barrier associated with early developmental age, which may contribute to the development of NEC, have not been thoroughly investigated. Herein, mucus barrier properties on excised ileum from 21do, 5do, N-S, N-S-LYS, and N-S-DHA rat pups were directly investigated using multiple particle tracking and time-lapse video microscopy to study passive particle diffusion and active microbe swimming in mucus. Chemical composition and structure of mucus were investigated for potential insight into observed differences in transport properties.

Particle diffusion was faster in mucus on immature (5do) tissue compared to 21do tissue, indicating a less effective barrier to passively diffusing entities. This finding was consistent with previous work that showed faster diffusion of 500 and 1000 nm carboxyl- modified particles through intestinal mucus from piglets (2-week old) relative to mucus from mature pigs (7–10 month old), which was also shown to contain more DNA^38^. The difference in transport properties between 5do and 21do mucus may be attributed to increased protein, mucin, and DNA content in the 21do samples. Specifically, an increase in DNA and mucin concentration has been shown to decrease particle diffusion and increase viscosity of the mucus gel^38,39^. The decrease in *E. coli* speed in 21do compared to 5do may also be due to these changes in mucus viscosity and composition. In the 21do mucus samples, there was a lower amount of sialic acid and fucose per mucin molecule; however, considering the higher amount of mucin in 21do samples, there was a significantly higher total amount of sialic acid and fucose. As mentioned previously, these mucin sugars play an important role in binding and inhibiting microbes from translocating through mucus to the underlying epithelium^40^ and may contribute to the observed decrease in microbe speed in 21do samples. In addition to mucin sugars, the amounts of other mucosal proteins (TFF3, IgG, and IgA) were investigated. 5do intestinal segments contained higher amounts of IgG, but lower amount of IgA compared to 21do. Mother’s milk consumed by 6do and 19do rat pups has been reported to have similar amounts of IgG and IgA^41,42^. IgG from milk is absorbed into the circulatory system^41^, and the rate of IgG absorption differs with developmental age resulting in higher IgG serum concentration in 19do compared with 6do pups^41^. This may at least partially explain the lower amount of IgG in 21do intestinal segments. In contrast, there are minimal differences in IgA absorption rate with developmental age^41^. In rats, maturity of the mucosal immune system and production of IgA occurs around 21 days^43^, which may explain the increase in IgA amount in 21do intestinal segments. IgA, which has been associated with a higher bactericidal effect on *E. coli* 0111 compared to IgG^44^, may have contributed to the decrease in microbe transport on 21do compared to 5do.

In agreement with composition analysis, a greater amount of mucus was observed in 21do compared to 5do, when the mucus layer in un-fixed intestinal samples was visualized by confocal microscopy. Although mucin analysis and confocal imaging suggested a higher amount of mucin in 21do tissues, electron micrographs indicated there was similar mucus coverage and microstructure on 21do and 5do tissues. The difference in observed mucus coverage may be attributed to sample preparation, i.e. loosely adherent luminal mucus being partially washed away during sample preparation for electron microscopy. Also, it is possible that the varied composition of the different mucosal barriers, including differences in DNA and potentially lipid content reflecting cellular debris, may impact fixation. Overall, decreased permeability of 21do mucus may be attributed to increased amounts of mucin, DNA, and IgA compared to 5do. These results indicate that mucus barrier properties depend upon developmental age, suggesting that permeability of mucus may be enhanced in prematurity, which inherently is associated with immaturity.

Premature pups delivered by cesarean section one day before scheduled birth were exposed to NEC stressors (i.e. formula feeding, hypoxia, and bacterial colonization)^45^. In these studies, approximately 40% of pups were sacrificed prior to day 5 due to signs of severe morbidity. The observed dependence of mucus barrier properties on developmental age suggests that early delivery by cesarean section may lead to vulnerability to microbial translocation and infection. Pups exposed to NEC stressors sacrificed at day 5 (N-S) had no evidence of disease and were used for mucus studies to exclude the confounder of intestinal necrosis. The mucus on N-S pups was less permeable (i.e. particle diffusion and microbe transport were slower) relative to 5do (healthy, full-term rat pups with free access to mother’s milk used as a model of intestinal immaturity). The strengthened mucus barrier may reflect protective changes in response to NEC stressors that aid in survival, and may be attributed to differences in mucus composition and structure. Although the amounts of total protein and mucin were similar, DNA amount in N-S was increased compared to 5do mucus, which may be due to hypoxia-induced apoptosis of epithelial cells^46^. This elevated DNA content may increase mucus viscosity and decrease particle transport rate and microbe speed in N-S compared to 5do. TFF3, IgG, and IgA amounts normalized to length were all lower in N-S than 5do, which may be attributed to N-S pups being fed formula instead of mother’s milk^32,47^. Decreased amounts of these protective and immunoregulatory proteins may contribute to a higher risk of developing NEC. Confocal images showed more lectin-stained mucus clustered around villi and SEM micrographs revealed smaller pores and aggregation of mucin fibers between villi compared with 5do. The decrease in mucus pore size may reduce the available volume for transport thus hindering particle diffusion and microbe speed in N-S samples.

Although differences in mucus barrier properties were observed for these N-S compared to healthy 5do pups, one limitation of this NEC animal model is the inability to directly compare diseased vs healthy NEC stressed pups since it requires sacrifice and histological analysis to diagnose NEC. It is not possible to study severely diseased NEC pups at developmental day 5 since sacrifice would have occurred prior to that time if animals showed signs of severe morbidity. Tissue obtained from diseased pups would be severely damaged by the disease process and changes to mucus properties would be obscured. The ileum was sampled as it is the most affected intestinal region in the NEC animal model, but the 6 cm of tissue collected was not adequate to allow pathological analysis, to assess potential evidence of NEC, along with mucus barrier analysis. Further, due to the cost and rigorous nature of this animal model, there are limitations in the number of pups that can be included in treatment groups. Finally, gender is difficult to determine for pups that are 5 days old or younger and thus was not identified in these studies.

The mucus barrier has been previously shown to be strengthened (i.e. decreased particle diffusion) by the presence of certain salts and lipids^31^, suggesting exogenous agents can have a significant impact on mucus barrier properties. In these studies, LYS and DHA were orally dosed to pups exposed to NEC stressors to determine their potential as prophylactic agents to strengthen the mucus barrier. LYS and DHA are absent or present in low concentrations in formula, but are known to be beneficial to gut health and mucosal barrier function^48–50^. In addition, we have previously observed that LYS and DHA decreased particle diffusion and *E. coli* mobility in porcine intestinal mucus (*ex vivo* data not published). LYS, an anti-microbial, is secreted by Paneth cells and kills microbes by cleaving glycosidic linkages in the bacterial cell wall^9,51^. Interestingly, NEC has been associated with a decrease in Paneth cell number and LYS concentration in Paneth cells^52,53^. Oral dosing of LYS to N-S pups (N-S-LYS) resulted in a less permeable mucus layer compared to N-S alone. The significantly decreased *E. coli* speed in N-S-LYS pups in spite of a significantly lower amount of sialic acid per mucin compared to N-S alone may be attributed to changes in mucus microstructure. Specifically, striated mucus in confocal images (Fig. 6) and large clusters of mucus between villi in SEM micrographs (Fig. 7) in N-S-LYS samples may be due to electrostatic interaction between positively-charged LYS and negatively-charged mucins^54^. The altered mucus structure may inhibit microbes from moving freely through mucus. The significant decrease in amine- and carboxyl- modified particle diffusion (but not PEG- modified particle diffusion) also seems to indicate a change in electrostatic interactions within mucus due to LYS exposure. Similar to LYS, oral dosing of DHA, a polyunsaturated fatty acid found in breast milk, was also found to impact the mucus barrier. DHA was investigated because (1) it is present in breast milk (0.06–1.4% of total fatty acids^55^), which has been shown to minimize the incidence of NEC in preterm infants^32^, and can be supplemented to formula (0.1–0.5% of total fatty acids^56,57^), (2) preterm infants have lower DHA levels in blood at birth compared to full-term infants^58^, and (3) oral dosage of DHA to mice has been shown to prevent microbe adhesion to the epithelium and minimize intestinal inflammation^50,59–62^. In our studies, N-S-DHA pups had a less permeable mucus layer as evidenced by significantly decreased microbe speed and charged particle diffusion rate when compared to N-S alone. However, there was no increase in the survival of N-S-DHA pups. There were minimal differences in mucus composition and structure of N-S-DHA compared to N-S alone, suggesting the altered transport properties are attributed to changes in the mucus microenvironment, i.e. electrostatic interactions or other compositional changes, not captured in our analyses. Overall, N-S-LYS or N-S-DHA resulted in a less permeable mucus layer compared to N-S alone, suggesting these potential prophylactic agents may be useful for inhibiting commensal microbes from penetrating mucus and invading the epithelium, thus minimizing disease onset and progression.

## Conclusion

Transport properties, chemical composition, and microstructure of mucus indicated mucus barrier properties vary with developmental age (21do vs 5do) and exposure to NEC stressors. Mucus from early developmental age rat pups (5do) had lower amounts of protein, mucin, and DNA, which may have altered mucus viscosity and binding interactions resulting in faster particle diffusion and microbe transport compared to 21do rat pups. The more permeable mucus barrier present in early developmental age (5do) compared with 21do pups suggests that intestinal immaturity may be associated with exposure of epithelium to microbes and luminal contents and thus contribute to the development of NEC. Interestingly, examination of intestinal samples of pups who survived exposure to NEC stressors over a 5 day time course revealed a less permeable barrier (i.e. slower particle diffusion and microbe transport) compared with tissue from non-stressed pups of the same age (5do) and was associated with a slightly aggregated mucus structure. These differences may be attributed in part to an increase in DNA due to hypoxia-induced apoptosis of epithelial cells. Importantly, our work suggests that changes in the mucus barrier may be important for survival under the stresses of formula feeding, hypoxia, and microbial pathogen exposure associated with NEC.

Oral dosage of LYS and DHA, which are components of breast milk, to N-S pups resulted in a less permeable mucus layer. Specifically, the transport of *E. coli* and charged particles was hindered, suggesting that the mucus barrier may be potentially strengthened to inhibit microbe transport and prevent translocation toward the epithelium. These changes were associated with a further increase in the survival of N-S-LYS pups compared to N-S alone, again suggesting that altered mucus may be important for protection when exposed to NEC relevant stressors. Further research into long-term dosing of LYS, DHA, or other potential prophylactic agents is crucial to investigate their potential to act as prophylactic treatments in preventing or minimizing NEC.

## Materials and Methods

### Neonatal rat necrotizing enterocolitis (NEC) model

All animal studies were approved by the University of Chicago Institutional Animal Care and Use Committee under Protocol No. 71557, and were performed in accordance with relevant guidelines and regulations. Hsd:Sprague Dawley^®^ SD^®^ rats were purchased from Envigo and used in experimental studies. Healthy control rat pups were delivered by normal vaginal birth and had free access to mother’s breast milk. Rat pups were sacrificed at postnatal age of 21 days (referred to as 21do), representing a relatively mature gut comparable to that of a 6 month old infant^63^. To model an immature infant gut, rat pups were sacrificed at postnatal age of 5 days (referred to as 5 do)^64^.

The experimental neonatal rat NEC model has been previously developed and characterized, confirming characteristics consistent with NEC disease in humans^65,66^. Briefly, neonatal Sprague-Dawley rats were delivered by cesarean section one day before scheduled birth. Every 3 hrs, pups were fed Esbilac puppy formula with an orogastric feeding catheter. The feeding volume began at 100 µl and was increased incrementally up to 250 µl over 5 days. Esbilac puppy formula was supplemented with 10^7^ CFU each of *Serratia marcescens*, *Klebsiella pneumonia*, and *Streptococci viridans*, generously provided by Dr. Tamas Jilling, once a day to colonize the intestine with microbes found at increased concentrations in NEC^16,67^.

Every 8 hrs, pups were exposed to hypoxic conditions (5% oxygen and 95% nitrogen) for 10 mins. Pups that survived exposure to NEC stressors were sacrificed at day 5 (referred to as NEC-stressed, N-S) and used for transport, chemical, and structural analysis of intestinal mucus. Pups were sacrificed earlier if they showed signs of severe morbidity and were not used in any experiments.

To test the impact of potential prophylactic treatments on mucus barrier properties of N-S pups, Esbilac puppy formula was supplemented with either egg white lysozyme (referred to as N-S-LYS) or docosahexaenoic acid (referred to as N-S-DHA) at every feeding (LYS and DHA from Sigma Aldrich). The amount of LYS and DHA was increased proportionally to the dosing volume – 0.37 and 0.82 mg of LYS, and 0.27 and 0.63 µl of DHA were dosed in 100 and 250 µl feeding volumes, respectively. Dosed concentrations were based on levels reported for human breast milk^55,68^.

After delivery by cesarian section, all rat pups were cleaned and allowed to stabilize at 37 °C. Following stabilization, pups were weighed and placed according to weight from heaviest to lightest. Pups were then sequentially and randomly assigned to different treatment groups, N-S, N-S-LYS, and N-S-DHA, which were identified as “Group A”, “Group B”, and “Group C”. The average weight of each treatment group was equal. Only the rat pup handler was able to identify the individual groups, and all assays and data analysis were run by individuals unaware of the treatment each group had received. Control samples, i.e. originating from 21 and 5 day old animals, were not blinded.

**Figure 9:**
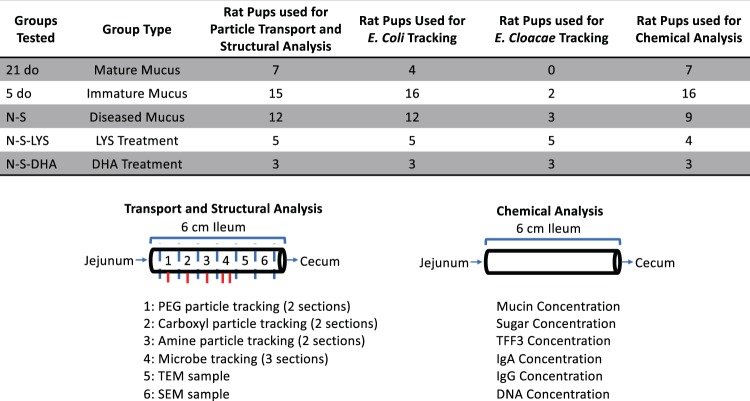
Table of groups tested identifying sample size with a schematic of ileal tissue allocation for transport, structural, and chemical analysis.

Fifteen rat pups were used in each NEC-stressed group (N-S, N-S-LYS, and N-S-DHA), with the N-S group tested in two independent experiments (30 pups total used). The pups that survived until day 5 were used for experiments. Following exposure to NEC stressors and prophylactic treatment, there were 21, 6, and 9 rat pups for N-S, N-S-DHA, and N-S-LYS groups. For control groups, there were fourteen 21 day old and twenty-one 5 day old pups. Tissue samples were used for analyses as indicated in Fig. 9. Immediately after sacrifice, approximately 6 cm of the ileum adjacent to the cecum was removed and maintained in phosphate buffered saline (PBS, Fisher BioReagents™) on ice. The ileum was selected for analysis since NEC-associated intestinal inflammation occurs primarily in the distal ileum^66,69^. The collected tissue was either divided and used for structural analysis as well as tracking of particles and/or microbes, or used for mucus collection and composition analysis, as described below.

### Particle and microbe preparation

Carboxyl- and amine- modified fluorescent polystyrene nanoparticles (200 nm diameter, FluoSpheres™, ThermoFisher Scientific) were used to probe mucus barrier properties^70^. PEG- modified nanoparticles were synthesized by conjugating amine-terminated polytheylene glycol (2000 MW, PEG-NH_4_, Laysan Bio, Inc.) to carboxyl- modified nanoparticles using 1-ethyl-3-(3-dimethylaminopropyl)-carbodiimide (EDC, Sigma Aldrich) crosslinking chemistry. Briefly, 20 mg PEG-NH_4_ and 250 µl carboxyl- modified particles were incubated for 15 mins in 50 mM 2-(N-morpholino) ethanesulfonic acid (500 µl MES, pH 6.5, Sigma Aldrich). Then, 10 mg EDC was added to the particle solution (pH 6.5) and incubated for 2 hrs. The reaction was quenched with 100 mM glycine (Sigma Aldrich) for 30 mins and dialyzed overnight using Slide-A-Lyzer® Mini Dialysis Devices with a 10 kDa cut off (ThermoFisher Scientific).

*Escherichia coli* MG1655 (*E. coli*, ATCC® 700926™) was transformed with a plasmid containing green fluorescent protein (GFP) and ampicillin resistance gene (gift from Marcia Goldberg, Harvard Medical School) using standard heat-shock protocol to obtain fluorescent *E. coli* for tracking^70^. Briefly, a 16 hr overnight culture of *E. coli* was diluted 1:100 in Luria Bertani (LB) broth (Fisher BioReagents™) and cultured for 2 hrs. The culture was centrifuged at 5000 rpm, 4 °C for 10 mins and the pellet was re-suspended in cold 100 mM calcium chloride (CaCl_2_, Sigma Aldrich) solution for 30 mins at 4 °C. The *E. coli* solution was then centrifuged and re-suspended in 2 ml of CaCl_2_. GFP-containing plasmid was added to 150 μl *E. coli* in CaCl_2_ and subjected to heat shock in a 42 °C water bath for 45 secs. The transformed cells were diluted in 1 ml LB broth and plated on ampicillin-containing LB agar (Fisher BioReagents™). After overnight culture, transformed GFP-expressing *E. coli* were selected, and frozen cultures were prepared in 20% glycerol solution (Sigma Aldrich) and stored at −80 °C. To prepare fluorescent *E. coli* for tracking experiments, 5 μl of frozen stock was point inoculated in the center of 0.3% LB agar motility plates. During overnight culture, microbes moved outward from the center and microbes (5 µl) at the edge of the colony were selected and cultured overnight in LB broth (6 ml) at 30 °C and 250 rpm. Microbe concentration was determined by diluting bacterial stock by 10^5^, plating 5 µl *E. coli* suspension on LB agar plates, and counting individual colonies after overnight incubation at 37 °C.

*Enterobacter cloacae* (*E. cloacae*, ATCC® 13047™, 5 µl) was cultured overnight in LB broth (6 ml) at 30 °C and 250 rpm and stained with SYTO^TM^ 9 green fluorescent nucleic acid stain (ThermoFisher Scientific) according to manufacturer’s protocol. Briefly, 3 μl of SYTO^®^ 9 solution was added to 1 ml of overnight culture of *E. cloacae* and incubated for 15 mins. Stained bacteria were then centrifuged at 3000 rpm for 5 mins and re-suspended in fresh LB broth to remove excess dye. Microbe concentration was determined by diluting bacterial stock by 10^5^, plating 5 µl *E. cloacae* suspension on LB agar plates, and counting individual colonies after overnight incubation at 37 °C.

### Tissue preparation for particle and microbe tracking

5do, 21do, N-S, N-S-LYS, and N-S-DHA rat pups were sacrificed individually directly prior to tracking to minimize tissue breakdown. A ~0.4 cm section of ileum collected as described above was cut open longitudinally, placed in a custom-made chamber (glass slide with rubber gasket), and any visible particulate or food matter was gently removed with forceps. Then, 0.5 μl particle solution (0.0025 wt% positively-charged amine-, negatively-charged carboxyl-, or neutrally-charged PEG- modified polystyrene beads in maleate buffer (MB)) or 0.5 μl microbe solution (~10^7^ colony forming units, *E. coli* or *E. cloacae*) was added to the exposed mucus surface. MB solution is composed of 100 mM Tris-maleate, 65 mM sodium chloride, 10 mM calcium chloride, 3 mM sodium azide, and 4 mM sodium hydroxide, at pH 6.5 (all chemicals from Sigma Aldrich). Tissues dosed with particles were incubated in a humid chamber at room temperature for 10 mins, while tissues dosed with microbes were incubated for 30 secs. An Olympus IX81 inverted wide field microscope with an electron multiplying charge coupled device (EM-CCD) Hamamatsu camera or an Olympus DSU spinning disk confocal with an EM-CCD Hamamatsu camera was utilized to take 10 sec videos with a frame rate of 30 frames per second, at 40× magnification. Particles and microbes were imaged on mucus layers closest to the tissue to prevent tracking in dosing medium or in areas of fluid drift. After tracking particles and microbes, Z stacks were collected to enable visualization of particle and microbe penetration between villi.

### Particle tracking analysis

A modified multiple particle tracking (MPT) MATLAB^®^ algorithm was used to calculate particle mean squared displacement (MSD) and effective diffusivity (D_eff_)^71^.

1 $${\rm{M}}{\rm{S}}{\rm{D}}={[{\rm{x}}({\rm{t}}+\tau )-{\rm{x}}({\rm{t}})]}^{2}+{[{\rm{y}}({\rm{t}}+\tau )-{\rm{y}}({\rm{t}})]}^{2}$$

2 $${{\rm{D}}}_{{\rm{eff}}}=\frac{{\rm{MSD}}}{4{\rm{\tau }}}$$

where x(t) and y(t) represent the particle coordinates at a given time and τ is the time scale. The MATLAB^®^ tracking algorithm was modified to identify 200 nm particles using the following parameters – particle size: 4 pixels, minimum particle intensity: 50, particle eccentricity: 0.1, and video image variables were changed to account for video image size (327×327 µm) and frame rate (30 frames/sec).

Anomalous particle diffusion was characterized by fitting the MSD vs time scale log-log plot with the following equation:

3 $$MSD=4{D}_{0}{{\rm{\tau }}}^{\alpha }$$

where D_0_ is the time-independent diffusion coefficient, τ is time scale, and α is the anomalous exponent, a parameter used to classify particle motion type. Particle movement was characterized according to the α-value, where 0 <α < 0.2 is considered immobile, 0.2 <α < 0.8 is sub-diffusive, and 0.8 <α < 1 is diffusive^20,29,72^. Trajectories of at least 300 particles were analyzed for each tissue, and mucus from 3 separate pups was utilized to account for mucus variability.

### Microbe tracking analysis

Microbial movement was analyzed and speed was quantified using the same MATLAB^®^ tracking algorithm utilized for particle tracking (microbe size: 4 pixels, minimum pixel intensity: 100, microbe eccentricity: (1). Speed was calculated using the following equation,

4 $$Speed=\frac{\sqrt{{[x(t+\tau )-x(t)]}^{2}+{[y(t+\tau )-y(t)]}^{2}}}{\tau }$$

where x(t) and y(t) represent the microbe coordinates at a given time, t, and τ is the time scale. Individual microbe trajectories were further characterized by calculating track linearity,

5 $$Track\,Linearity\, \% =\frac{DIS}{LEN}\,\ast \,100 \% $$

where DIS is the distance from the start to the end of the track, and LEN is the length along the entire track^73^. The track linearity (TL) was used to classify the movement type for each individual trajectory as Rotating (TL < 30%), Curvilinear (30% > TL > 70%), or Linear (TL > 70%).

### Confocal imaging

Tissue samples were opened longitudinally to expose the mucosa and stained with 0.5 μl of 1 mg/ml wheat germ agglutinin (ThermoFisher Scientific), which binds to sialic acid and N-acetylglucosaminyl sugars. Tissue samples were also stained with 0.5 μl of 5 mg/ml 4′,6-Diamidino-2-Phenylindole, Dihydrochloride (DAPI, ThermoFisher Scientific) or 5 mM CellTrace^TM^ Bodipy^TM^ TR Methyl Ester (ThermoFisher Scientific) to visualize and identify nuclei and endomembranous organelles (i.e. endoplasmic reticulum and Golgi apparatus) of intestinal epithelium, respectively. Confocal Z stacks were obtained using a FV1000 Laser Scanning Confocal Microscope. ImageJ was used to create maximum intensity projection images from Z stacks.

### Mucus collection for biochemical analysis

Mucus was collected from the intestine of 5do, 21do, N-S, N-S-LYS, and N-S-DHA rat pups. Approximately 6 cm of ileum adjacent to the cecum was removed as described above. Mucus was collected by using a 30 gauge needle (0.5 inch in length, BD PrecisionGlide^®^) to inject 40 µl of 10 mM N-acetylcysteine (Sigma Aldrich), which cleaves disulfide bonds between mucins and aids in removal of the mucus layer^74^, into the intestinal lumen. The tissue was then massaged with forceps, flushed with 200 µl PBS using a 30 gauge needle, and gently squeezed with forceps to push mucus from the lumen. 50 µl of protease inhibitor cocktail (cOmplete™, Roche) was added to approximately 400 µl of collected mucus to prevent protein degradation. Collected mucus was stored at −20 °C until analysis.

### Characterization of mucus proteins

Thermo Scientific^TM^ Pierce^TM^ Bicinchoninic Acid (BCA) Protein Assay was used to quantify total protein amount. Briefly, 25 µl of mucus sample was added in triplicate to a 96-well plate. Then, 200 µl of the working reagent (50 parts of BCA Reagent A with 1 part of BCA Reagent B) was added to each well and incubated at 37 °C for 30 mins. Absorbance was measured at 562 nm using a BioTek Powerwave XS spectrophotometer. A standard curve of bovine serum albumin (0–2000 µg/ml) was used to calculate sample protein amount.

Mucin amount was quantified using an alcian-blue assay^70,75^. Each sample or standard (0–250 µg/ml of mucin from bovine submaxillary glands, Sigma Aldrich) was added (100 µl) in triplicate to a 96 well plate followed by 33.3 µl of alcian blue (Sigma Aldrich). The plate was incubated on an orbital shaker for 2 hrs at room temperature and then centrifuged at 1500 × g for 30 mins. The plate was gently inverted on paper towels to blot the liquid from the wells. The wells were washed three times to remove excess alcian blue stain by adding 100 µl of wash buffer (290 ml 70% ethanol, 210 ml 0.1 M acetic acid, 1.2 g magnesium chloride, all chemicals from Sigma Aldrich), centrifuging for 10 mins at 1500 × g, and removing supernatant. After the third wash, 100 µl of 10% sodium dodecyl sulfate (Sigma Aldrich) was added to each well. The plate was placed on an orbital shaker for 30 mins to dissolve the mucin. Absorbance was measured at 620 nm using a BioTek Powerwave XS spectrophotometer.

The amounts of Immunoglobulin G (IgG), Immunoglobulin A (IgA), and trefoil factor 3 (TFF3) collected from N-S, 5do, and 21do pups were determined using enzyme-linked immunosorbent assay (ELISA, MyBioSource). ELISA assays were run according to manufacturer’s protocol. Briefly, 100 µl of standard or sample was loaded into an ELISA plate and incubated for 2 hrs at 37 °C. The liquid was removed, 100 µl of detection reagent A was added, and the plate was incubated for 1 hr at 37 °C. Each well was washed 3 times with 350 µl wash solution. 100 µl of detection reagent B was added to each well and incubated for 30 mins at 37 °C. The wells were washed 5 times with 350 µl wash solution. Then, 90 µl of substrate solution was added to each well, followed by incubation for 20 mins at 37 °C, and addition of 50 µl of stop solution to stop the reaction. Absorbance was measured at 450 nm using a BioTek Powerwave XS spectrophotometer. Standard curves were used to calculate unknown IgG, IgA, and TFF3 amounts.

### Characterization of mucin sugars

Two mucin sugars that are essential for microbial binding, fucose and sialic acid, were quantified^40^. Fucose was quantified using Megazyme Assay Kit. In a 96 well plate, 200 µl distilled water, 10 µl standard (0.1–10 µg/well) or sample solution (run in triplicate), 40 µl of buffer 1 solution, and 10 µl of nicotinamide-adenine dinucleotide phosphate (NADP+) were added. The plate was gently mixed on an orbital shaker for 5 mins and then 2 µl of L-fucose dehydrogenase (L-FDH) was added. The absorbance was measured at 340 nm using a BioTek Powerwave XS spectrophotometer.

Sialic acid amount was determined using Abcam Sialic Acid (NANA) Assay Kit. Briefly, 50 µl of standard (0–10 nmol/well) or sample was added to each well in triplicate. Then 50 µl of reaction mix (44 µl assay buffer, 2 µl sialic acid converting enzyme, 2 µl sialic acid development mix, and 2 µl sialic acid probe) was added to each well and incubated for 30 mins protected from light. Absorbance of each well was measured at 570 nm.

### DNA quantification

CyQUANT® cell proliferation assay kit (ThermoFisher Scientific) was used to measure DNA. In a 96 well plate, 100 μl of standard or sample and 100 μl of CyQUANT® GR dye was added and incubated for 5 mins at room temperature. The fluorescence of the samples (480/520 ex/em) was measured using an EnSight^TM^ multimode plate reader (PerkinElmer, Inc.). Bacteriophage λ DNA was used to make a standard curve, with standard DNA concentrations ranging from 0–1000 ng/ml.

### Electron microscopy

To prepare samples for transmission electron microscopy (TEM), approximately 0.3 cm of ileum adjacent to the cecum was collected and fixed with perfluorohexane/1% osmium tetroxide solution^36,37^ (chemicals from Sigma Aldrich) for 2 hours on ice and then placed in perfluorohexane for 2 days at 4 °C. Samples were exchanged in 100% ethanol (Sigma Aldrich), embedded in Spurr low-viscosity resin (Electron Microscopy Sciences), and polymerized for 24 hrs at 70 °C. The intestinal cross-section was thin sectioned using an ultramicrotome (Reichert-Jung Ultracut E), mounted on copper-coated carbon grids (Electron Microcopy Sciences), and imaged using a JEOL JEM-1010 transition electron microscope.

To prepare samples for scanning electron microscopy (SEM), approximately 0.75 cm of ileum about 0.7 cm prior to the cecum was collected and divided into two pieces. Samples were cut open longitudinally and immersed in perfluorohexane/1% osmium tetroxide solution or Carnoy’s fixative^36,37^ (10% glacial acetic acid, 30% chloroform, 60% ethanol, chemicals from Sigma Aldrich) for 2 hours at 4 °C. Samples were exchanged in 100% ethanol, critical point dried using Samdri^®^-PVT-3D drier, and sputter-coated with 10 nm gold using Cressington Sputter Coater 208HR. Scanning electron micrographs were taken using a Hitachi S-4800 field emission scanning electron microscope operated at 2 kV.

### Statistical analysis

Particle and microbe tracking experiments were completed using tissue from at least 3 different rat pups, with two tissue sections per particle (amine-, carboxyl-, and PEG- modified) and microbe (*E. coli*, *E. cloacae*) for each pup. For each particle or microbe type, a total of at least 300 tracks were analyzed for each pup. Structural (i.e., scanning electron, transmission electron, and confocal microscopy) and chemical analyses were also completed using tissue from at least 3 different rat pups to account for mucus and tissue variability. Chemical data was normalized to collected mucus volume and tissue length. Tracking data for all collected particle or microbe tracks are presented as an overall mean with standard error of the mean (SEM), and chemical analysis plots are presented as a mean with standard deviation (SD). The percent particle (immobile, subdiffusive, and diffusive) and microbe (linear, curvilinear, and rotating) motion type for each individual tissue was obtained, and the average of these means was presented for each group, with SD representing the deviation between the means of each tissue. ANOVA was used to determine significance between groups with α = 0.05.

## Supplementary information

Supplementary Information.

## Data Availability

The datasets generated during and/or analysed during the current study are available from the corresponding author on reasonable request.
