# Supplementary material for: Impact of Developmental Age, Necrotizing Enterocolitis Associated Stress, and Oral Therapeutic Intervention on Mucus Barrier Properties
Source: Sci Rep. 2020 Apr 21;10:6692. doi: 10.1038/s41598-020-63593-5 (PMC7174379; doi:10.1038/s41598-020-63593-5)
Supplement: Supplementary file 1 — Supplementary Information. [file 41598_2020_63593_MOESM1_ESM.pdf]

Impact of Developmental Age, Necrotizing Enterocolitis Associated Stress, and Oral Therapeutic Intervention on Mucus Barrier Properties

Jaclyn Y. Lock[1], Taylor L. Carlson[2], Yueyue Yu[3], Jing Lu[3], Erika C. Claud[3,4], Rebecca L. Carrier[2*]

[1]Department of Bioengineering, Northeastern University, Boston, Massachusetts

[2]Department of Chemical Engineering, Northeastern University, Boston, Massachusetts

[3]Department of Pediatrics, Section of Neonatology, University of Chicago, Chicago, Illinois

[4]Department of Medicine, Section of Gastroenterology, University of Chicago, Chicago, Illinois

*Corresponding Author, Email: Rebecca@coe.neu.edu

Supplemental Information:

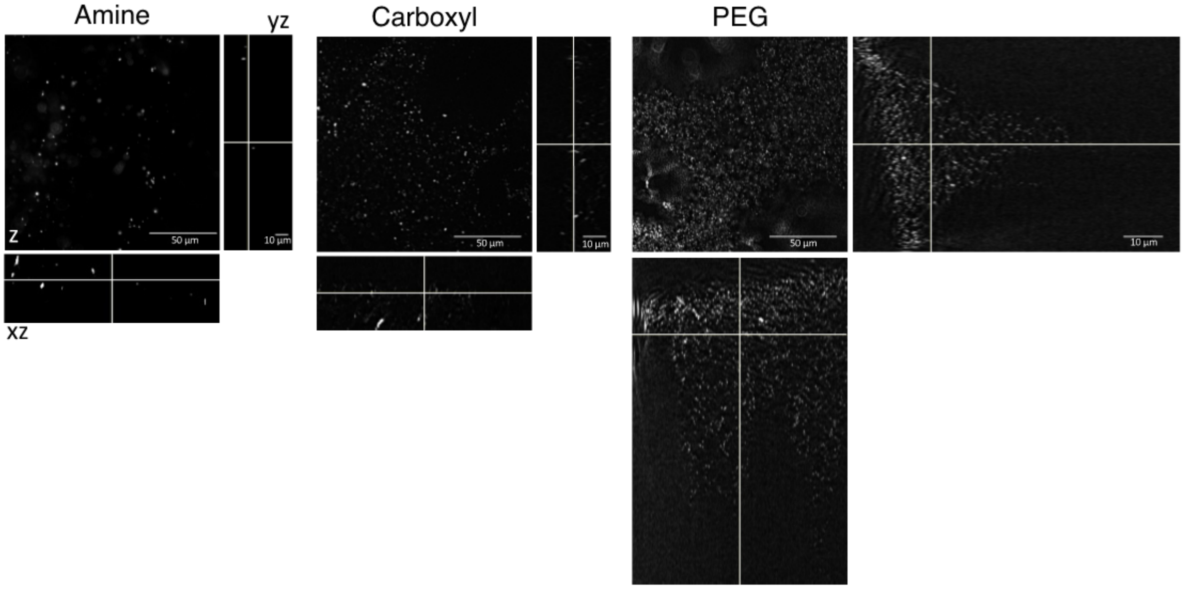

Supplementary Fig S1: Representative Z stack of amine-, carboxyl-, and PEG- modified particles in mucus 10 mins after adding particles to the mucosal surface. Images show a single Z slice (z) with corresponding orthogonal views (xz and yz). Intersection of lines designate the point in the z-stack that is being projected in the orthogonal view.

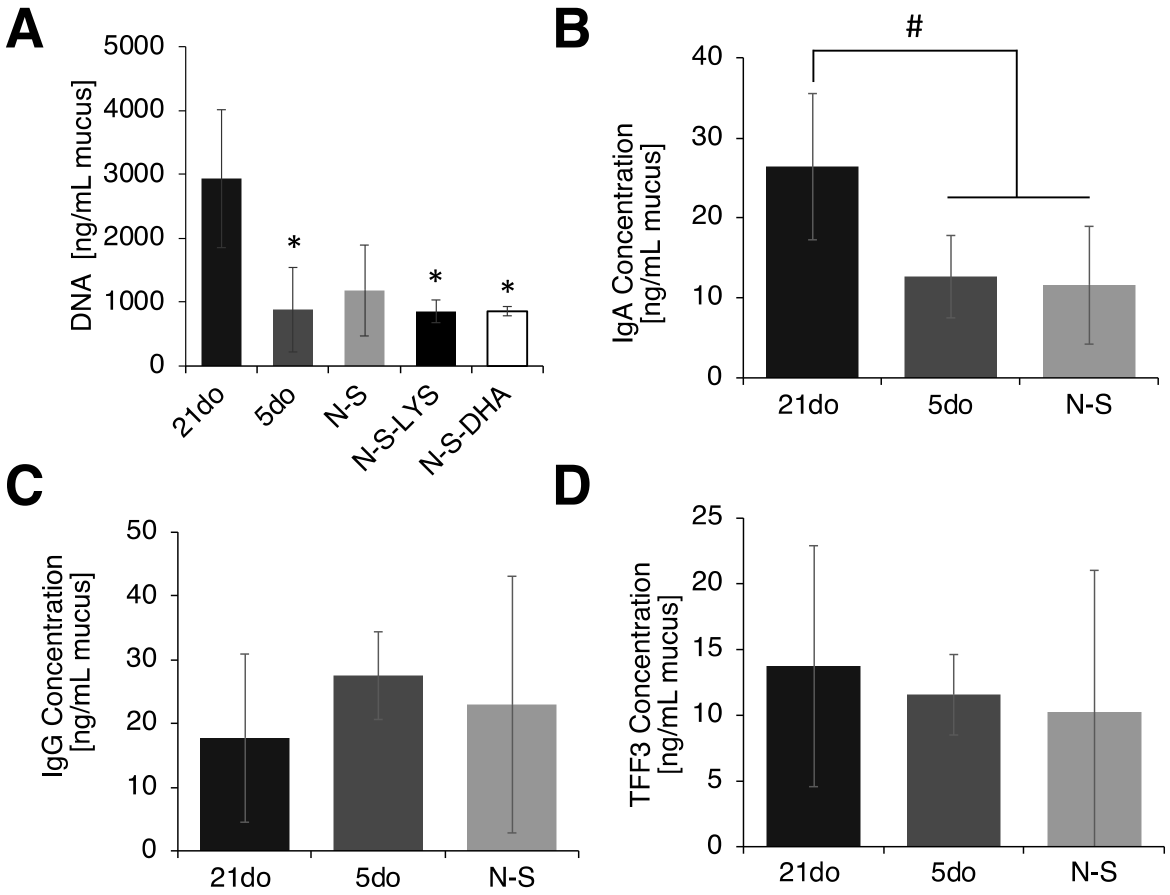

Supplementary Fig S2: **(A)** DNA amount per volume of mucus collected from 21do, 5do, NEC-stressed (N-S), NEC-stressed and orally dosed with LYS (N-S-LYS), and NEC-stressed and orally dosed with DHA (N-S-DHA) tissue. **(B)** Immunoglobulin A (IgA), **(C)** Immunoglobulin G (IgG), and **(D)** Trefoil factor 3 (TFF3) amount per volume of mucus collected from 21do, 5do, and N-S pups. ANOVA was used to determine significance in (A) * $p < 0.05$ comparing 21do to 5do, N-S-LYS, and N-S-DHA, and in (B) # $p < 0.05$ comparing 21do to 5do and N-S.
